# Clinical value of CVP+VIVC in predicting fluid resuscitation in patients with septic shock

**DOI:** 10.4314/ahs.v23i3.52

**Published:** 2023-09

**Authors:** Haitao Zhang, Chang Liu, Aiping Cao, Qiong Hang

**Affiliations:** 1 Department of Critical Care Medicine, Xinrui Hospital, Xinwu District, Wuxi, China; 2 Department of Critical Care Medicine, Guangde People's Hospital, Xuancheng, China; 3 Xinrui Hospital, Xinwu District, Wuxi City. 197 Zhixian Road, Hongshan Town, Xinwu District, Wuxi City, Jiangsu Province

**Keywords:** Septic shock, fluid resuscitation, CVP, VIVC

## Abstract

**Objective:**

To explore the clinical value of central venous pressure (CVP) + inferior vena cava respiratory variability (VIVC) in fluid resuscitation in spontaneously breathing patients with septic shock.

**Methods:**

In retrospective observational study, during October 2019 to December 2021, 145 patients with septic shock treated in our hospital were enrolled by the method of observational study. According to the change rate of cardiac output (ΔCO) ≥15% or ΔCO<15% after 30 minutes, they were assigned into volume-responsive and volume-unresponsive group depending early fluid resuscitation in sepsis. The clinical value of combination of CVP and VIVC in predicting fluid resuscitation in patients with septic shock was compared.

**Results:**

The CVP of the study group was higher at 12h and 24h after fluid resuscitation, and the VIVC level of the study group at 6h, 12h and 24h after fluid resuscitation was higher (P<0.05). Pearson correlation analysis indicated that CVP, and VIVC levels were noticeably correlated with fluid resuscitation in patients with septic shock (P<0.05). The area under curve (AUC) of receiver operating characteristic curve (ROC) of CVP for predicting fluid resuscitation in septic shock patients was 0.694 and the cut-off value was 0.932, the sensitivity was 46.9%, and the specificity was 87.5%. VIVC predicted fluid resuscitation in septic shock patients with an AUC of 0.776, which was a cut-off value of 0.688, a sensitivity of 50.0%, and a specificity of 90.0%. Combination of CVP and VIVC predicted fluid resuscitation in septic shock patients with an AUC of 0.948, which was a cut-off value of 1.420, a sensitivity of 90.6%, and a specificity of 87.5%.

**Conclusion:**

Combination of CVP and VIVC may have a good effect on the evaluation of volume responsiveness in patients with septic shock, which is better than single CVP and VIVC. Combination of CVP and VIVC can be adopted to predict fluid responsiveness volume responsiveness in septic shock patients, which is of great significance for guiding clinical fluid responsiveness therapy.

## Introduction

Sepsis is a common and extremely dangerous disease. The incidence of sepsis has increased considerably due to the development of various invasive medical treatments. There are approximately one million new cases of sepsis worldwide each year, making it one of the leading causes of death in intensive care units 1-2. As a result, it is very important to find ways to treat patients with septic shock effectively 3. During septic shock, patients are characterized by an imbalance of hemodynamic state. The peripheral systemic circulatory resistance is reduced but the cardiac output is mostly normal or increased 4. For patients with septic shock, early, reasonable and effective fluid resuscitation is the key to improving hemodynamic imbalance 5.

The evaluation index of volume response has been a hot spot of study for domestic and overseas scholars. The static pressure index, which indirectly describes the level of cardiac preload through pressure, which is only applicable to the volume assessment of hypovolemic patients. Since the relationship between pressure and volume is not a simple linear relationship, its accuracy and reliability are not high 6. The static volume index can reflect the cardiac preload more accurately than the pressure index. The same preload may be located on the ascending phase of the Frank-Starling curve or on the plateau branch for different myocardial compliance and patients. Assessing volume responsiveness is to distinguish patients on the ascending phase of the Frank-Starling curve 7. From a physiological point of view, dynamic indicators are preferable to static indicators. In 2018, the latest guidelines for the treatment of sepsis recommend the use of dynamic indicators to predict volume responsiveness 8. Dynamic indicators include passive leg raising test, volumetric loading test, variation in volume per beat and systolic blood pressure before and after fluid replacement, and variation in pulse pressure 9.

Hemodynamic monitoring has converted from a static to a dynamic indicator, providing real-time guidance for clinical care. Central venous pressure (CVP)-guided fluid resuscitation, pulse-indicated continuous cardiac output monitoring and pulmonary artery float catheters have long been utilized to monitor hemodynamics in the critical ill. Its application is limited by its imprecision and invasiveness. CVP is similar to right ventricular pressure and may be used as a predictor of preload. CVP may be influenced by peripheral blood pressure, cardiac function, airway pressure and central venous cannulation technique. Meanwhile, puncture may lead to hematoma and infectious complications. In addition, there are limitations as patients with coagulation disorders may experience heavy bleeding and worsen their condition 10.

The inferior vena cava (IVC) is a volume vessel with good compliance in the human body. Patients in septic shock have reduced effective circulating blood volume, collapsed inferior vena cava diameter and an increase in diameter with respiratory variability. The inferior vena cava respiratory variability (VIVC) can therefore be adopted to detect the volume status of patients in septic shock and thus guide fluid resuscitation efforts 11. In addition, this index has the advantages of non-invasive, simple, repeatable. There is a positive correlation between inferior vena cava diameter and CVP, while there is a negative correlation between VIVC and CVP. It has been shown in some studies that the combined application of CVP and VIVC is more accurate in predicting the volume responsiveness of patients 12-13. Therefore, when patients are in septic shock, it is recommended to use both CVP and VIVC to evaluate the volume status of patients because it is simple, timely and reliable, so it has become an important tool in the field of critical medicine recently. Despite advancements in medical technology, the prevalence of sepsis and its associated complications is growing. The septic shock and multiorgan failure syndromes continue to be a therapeutic difficulty for both primary care physicians and intensivists.

Therapy is still primarily support-based and mortality disproportionately rises with the development of organ failure, underlining the importance of the necessity of prevention. However, there are currently few similar studies according to our current acknowledge, which is difficult to accurately explain the clinical value of CVP+VIVC prediction in fluid resuscitation of patients with septic shock based on a few literatures. Our study is one of the few to use CVP+VIVC to predict sepsis. Under this background, further scientific research is needed to draw more scientific and accurate conclusions and help clinicians better judge the changes of patients' conditions. In light of this, the current study examined whether CP+-VIVC prediction for fluid resuscitation in septic shock has clinical utility.

## Patients and methods

### General information

**Inclusion criteria:** The patients met the diagnostic criteria for sepsis in the 2016 Third Edition of the International Consensus on Definition of Sepsis and Septic Shock, and the criteria for fluid resuscitation also referred to this document 14. Followed by clinical manifestations of septic shock, systolic blood pressure was <90mmHg (11.97kPa), or mean arterial pressure (MAP) was <60mmHg (7.98kPa), arterial blood pressure decreased by more than 40mmHg (5.32kPa) compared with the basic level; 2) there were clinical manifestations of insufficient perfusion of tissues and organs, such as lactic acidosis, anuria or oliguria, or accompanied by changes in the state of consciousness, etc.; 3) vasoactive drugs were required to maintain stable blood pressure or blood lactate concentration < 2mmol/ L.

### Exclusion criteria

(1) Patients with severe heart failure and structural heart disease; 2) patients who have had chronic kidney disease; 3) patients with recent acute coronary syndrome, or those who had undergone cardiopulmonary resuscitation and electrical cardioversion; 4) pregnant women; 5) patients were younger than 18 years old; 6) patients who quit halfway due to changes in the condition and did not complete the 30-min volume resuscitation; 7) patients with severe mental disorders and cognitive dysfunction.

In this study, 145 spontaneously breathing patients with septic shock cured in our hospital were enrolled by the method of retrospective observational study during October 2019 to December 2021. Cardiac output (CO) 15 was measured via a phase-controlled probe with a frequency of 1-5MHz to connect the electrocardiogram when the patient took the supine position. In the hands of an experienced intensivist, critical echocardiography can accurately measure CO according to previous study 15. First, the probe marking point was pointed to the side of the head to obtain the parasternal left ventricular long axis section, and the left ventricular end-diastolic diameter and left ventricular end-systolic diameter were measured. Based on the ultrasound software's built-in functions 16, left ventricular end-diastolic volume (LEDV) and left ventricular end-systolic volume (LESV) were calculated. Then, the OC was obtained according to stroke volume (V) = LEDV-LESV, CO= heart rate (HR) * stroke volume (SV). According to the change rate of cardiac output (DCO) ≥15% or □CO<15% after 30 minutes, they were assigned into volume-responsive group (n=80) and volume-unresponsive group (n=65). All patients signed informed consent forms after our hospital's medical ethics committee approved the study. Ethics committee approval number (20190013).

### Treatment methods

This study was conducted from the time the patient was diagnosed with septic shock until 28 days after treatment, when the patient was admitted to the ICU or after admission to the ICU. All patients underwent early fluid resuscitation. For resuscitation, one should give crystalloids at a dose of 30 mL/kg of ideal body weight as early as possible, typically within the first 3 hours, with rapidly administer a minimum of 30 mL/kg crystalloid intravenously early in the resuscitation period 17. The fluid resuscitation would spend 10 to 15 minutes.

Determination of CVP: During the procedure, the patient was placed in the supine position and the right internal jugular or subclavian vein was punctured. The two ports of the central venous catheter were inserted, with the intersection of the right axillary midline and the fourth intercostal space as the zero point, and readings were taken at the end of the entire respiratory cycle.

VIVC determination: During the procedure, the patient was positioned supine, and the longitudinal section of the inferior vena cava was measured by two-dimensional ultrasound. In addition, the diameter of inferior vena cava at the end of inspiration (Dinsp). Three times were measured, and the average value was calculated. VIVC=(-Dinsp-Dexp)/Dexpx100%. The CVP and VIVC of the two groups of patients were measured before and after 6h, 12h, 24h of fluid resuscitation.

### Data collection

The general data of the patients were collected on the same day or the next day after admission, including the general clinical data of each enrolled patient, acute physiology and chronic health score II within 24 hours of admission (APACHE II), Sequential Organ Failure Assessment (SOFA), the number of lung infections, the number of digestive system infections, the number of urinary system infections, the number of cardiovascular diseases, and the number of assisted ventilation treatment.

While inter-observer variation can rarely be avoided, it can be minimized by limiting the number of observers for each variable and by reviewing repeated measurements each time to capture and correct for drift in the measurers during data collection.

### Statistical analysis

The test data were statistically analysed by SPSS22.0 software. An independent sample t-test was used to compare the data between the two groups with normal distribution and homogeneity of variance ( ±s). The counting data and continuous data were presented by [n (%)], and the comparison was carried out by χ 2 test. Mann-Whitney U test was used to analyse non-parametric data. The correlation between the two normal distribution indexes was analysed by Pearson correlation analysis. The AUC of ROC was adopted to assess the predictive value of CVP and VIVC combination in fluid resuscitation in patients with septic shock. P<0.05 was found to be statistically significantly different.

## Results

### Comparison of general data

Comparison of gender, age, body mass index, APACHE II score, SOFA score, the number of lung infections, digestive system infections, urinary system infections, cardiovascular diseases, diabetes mellitus, and assisted ventilations. There were no significant differences in the number of patients treated, CVP, VIVC or years of education(P>0.05). Table 1 shows the results of all the data analysis.

**Table 1:** The general data of two groups of patients

Group	Capacity response group (n=80)	Capacity non-response group (n=65)	χ*2*	*P*
Gender	38/42	31/34	0.000	>0.05
Age (years)	56.95±2.11	56.55±2.53	1.038	>0.05
Body mass index (kg/m^2^)	24.11±2.14	24.02±2.11	0.253	>0.05
APACHE II score	27.48±2.95	27.49±2.21	0.022	>0.05
SOFA score	15.49±2.35	15.91±2.35	1.070	>0.05
Pulmonary infection	10 (12.50)	11 (16.92)	0.566	>0.05
Digestive system infection	4 (5.00)	8 (12.31)	2.522	>0.05
Urinary system infection	6 (7.50)	9 (13.85)	1.557	>0.05
Cardiovascular diseases	9 (11.25)	6 (9.23)	0.157	>0.05
diabetes	8 (10.00)	6 (9.23)	0.024	>0.05
Auxiliary ventilation therapy	17 (21.25)	8 (12.31)	2.009	>0.05
CVP (mmHg)	8.45±2.55	8.49±2.56	0.093	>0.05
VIVC (%)	21.91±3.56	21.66±3.54	0.421	>0.05
Number of years of education (years)	10.34±1.27	10.40±1.29	0.281	>0.05

### CVP and VIVC levels at 6 h, 12 h and 24 h after fluid resuscitation

There exhibited no difference in 6h CVP after fluid resuscitation (P<0.05). The CVP of the study group was higher at 12h and 24h after fluid resuscitation, and the VIVC level at 6h, 12h and 24h after fluid resuscitation was higher (P<0.05). Table 2 shows the results of all the data analysis.

**Table 2:** CVP and VIVC levels between the two groups at 6 h, 12 h and 24 h after fluid resuscitation[*x̅*±s]

Group	N	CVP (mmHg)			VIVC (%)		
		After recovery 6h	After recovery 12h	After recovery 24h	After recovery 6h	After recovery 12h	After recovery 24h
Capacity response group	80	8.95±2.46	8.61±1.25	8.25±2.21	19.58±2.53	15.18±2.39	14.91+2.44
Capacity non-response group	65	8.58±2.35	9.24+2.13	9.82±2.11	20.91+2.21	20.05+2.15	19.48±2.35
*t*		0.918	2.217	4.341	3.329	12.759	11.402
*P*		>0.05	<0.05	<0.001	< 0.05	< 0.01	<0.01

### The relationship between CVP, VIVC levels and 24-hour fluid resuscitation in patients with septic shock

Pearson correlation analysis indicated that the levels of CVP and VIVC were noticeably correlated with 24-hour fluid resuscitation in patients with septic shock (P<0.05). Table 3 shows the results of all the data analysis.

**Table 3:** Correlation analysis between CVP, VIVC levels and fluid resuscitation in patients with septic shock

Group	*r*	*P*
CVP	0.842	<0.05
VIVC	-0.596	<0.05

### Logistic regression analysis of multiple factors affecting fluid resuscitation in patients with septic shock

Multivariate logistic regression analysis indicated that CVP and VIVC were risk factors for fluid resuscitation in patients with septic shock (P<0.05). Table 4 shows the results of all the data analysis.

**Table 4:** Multivariate logistic regression analysis of fluid resuscitation in patients with septic shock

Group	b	S. E	Chi-square value	P	OR	95% CI for OR
CVP	1.241	0.153	65.790	0.000	3.459	2.563-4.669
VIVC	-1.099	0.231	26.622	0.000	0.333	0.219-0.506

### ROC of fluid resuscitation predicted by CVP+VIVC in patients with septic shock

**Figure 1:**
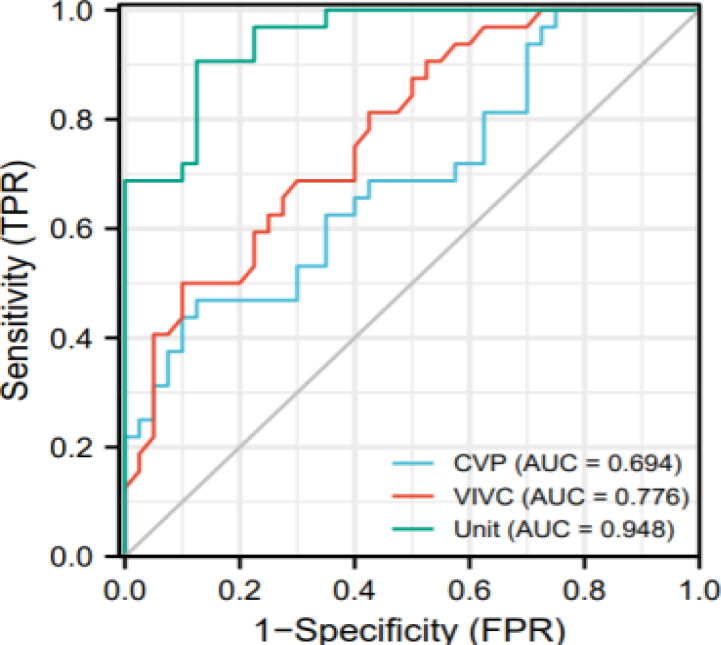
Combination of CVP and VIVC predicts ROC curve of fluid resuscitation in patients with septic shock

The predictive AUC of CVP for fluid resuscitation in patients with septic shock was 0.694, with a sensitivity of 46.9%, specificity of 87.5% and a cut-off value of 0.932. The AUC of VIVC in predicting fluid resuscitation in septic shock patients was 0.776, the cut-off value was 0.688, the sensitivity was 50.0%, and the specificity was 90.0%. The AUC of CVP and VIVC combination for predicting fluid resuscitation in patients with septic shock was 0.948, the cut-off value was 1.420, the sensitivity was 90.6%, and the specificity was 87.5% (P<0.05). All data results are shown in Figure 1 and Table 5.

**Table 5:** ROC of predicting fluid resuscitation in patients with septic shock

predictor variable	Cut-off Value	Sensitivity	Specificity degree	Positive predictive value	Negative predictive value	Yoden index
CVP	0.932	0.469	0.875	0.750	0.673	0.344
VIVC	0.688	0.500	0.900	0.800	0.692	0.400
CVP and VIVC	1.420	0.906	0.875	0.853	0.921	0.781

## Discussion

Sepsis is characterized by high morbidity, high mortality and high cost of treatment 18. As sepsis deaths have become a global medical problem each year 19. As a consequence of septic shock, fluid management is inseparable from the patient's condition. Therefore, it is essential to determine whether volume load will improve the patient's insufficient perfusion. Indicators for the evaluation of volumetric response have been a hot and difficult area of research. A wide variety of clinical indicators are available, ranging from blood pressure, heart rate, urine output, consciousness, and skin perfusion, to pressure load evaluation indicators, such as the CVP and pulmonary artery wedge pressure. Then there are indicators of volumetric load assessment, like left/right/total ventricular end-diastolic volumes, and a range of indicators based on cardiopulmonary interactions 20-22. The evaluation indicators of competence responsiveness have undergone a transition from static to dynamic, from qualitative to quantitative, and from invasive to non-invasive. Volume load generally refers to the preload of the heart. In clinical practice, fluid replacement is often used to increase cardiac preload 23.

Volumetric reactivity refers to the ability to increase SV or CO after fluid resuscitation, generally with an increase greater than or equal to 10% and 15% as capacity reactivity 24. Precisely, volumetric loading a volumetric responsiveness are not the same concept. The purpose of our study of volume responsiveness was to improve organ tissue ischemia and hypoxia while avoiding volume overload, increasing mortality and hospitalization time. More attention should be paid to the cardiac function to evaluate the volume responsiveness. If both the left and right ventricles are in the ascending phase of the Frank-Starling curve, there is volume responsiveness as OC will increase with increased cardiac preload after fluid resuscitation 25. A ventricle is in the plateau phase of the Frank-Starling curve, and even increasing cardiac preload with fluids does not noticeably increase cardiac output. Previous study has found that patients with volume responsiveness account for only 40-50% of hemodynamically unstable patients 26. As a result, excessive fluid replacement can cause pulmonary oedema and heart failure, affecting the metabolism and oxygenation of organs and tissues, aggravating the patient's condition and ultimately leading to increased mortality 27.

CVP usually can be measured directly by inserting a central venous catheter 28. It is known that the initial length of ventricular muscle is determined by the amount of ventricular end-diastolic filling. Preload and end-diastolic volume of the ventricles are equivalent 29. Because there is a good correlation between EDV and intraventricular pressure in a certain range, and intraventricular pressure measurement is more convenient than volume measurement, ventricular end-diastolic pressure is often used to reflect preload 30. In normal subjects, end-diastolic intra-atrial pressure is almost equal to intraventricular pressure. The magnitude of the CVP can reflect the pressure in the right atrium, and CVP is relatively easy to measure and can be used as a proxy to approximate preload. It is generally believed that the normal value range of CVP is between 5 and 10cmH2O 31.

If CVP < 5cm H2O, it indicates right atrial filling or insufficient blood volume; if CVP > 15cm H2O, it indicates excessive contraction of venous bed or cardiac insufficiency or increased pulmonary vascular resistance; if CVP is greater than 20cm H2O, it represents congestive heart failure 32. However, the pressure index is not an index of volume and the relationship between pressure and volume is not simply a linear correlation. The study has proved that CVP is not an accurate and reliable index for predicting liquid reactivity, and the position of CVP in predicting volumetric reactivity has been challenged 33-34. The main factors influencing CVP monitoring are patient position, intrathoracic pressure, mechanical ventilation, and the accuracy of the measurement method. In the presence of a large pericardial effusion, high abdominal pressure and increased extracardiac pressure, CVP increases, venous return decreases and cardiac preload decreases. Secondly, ventricular compliance can affect the corresponding relationship between CVP and preload. The curve of EDV with pressure is closely related to ventricular compliance, but ventricular compliance is not constant. The relationship between EDV and pressure is neither linear nor the only way 35. It is a fact that when EDV is increased, CVP will increase as atrial compliance decreases; the same CVP can correspond to various EDVs, depending on the actual atrial compliance in other words. Ultimately, variations in CVP may be caused by changes in cardiac function and/or venous return, so a single CVP value may correspond to multiple cardiac functional states and venous return 36-37. The predictive effect of single CVP value on volumetric reactivity is limited, and the predictive value of CVP change on volumetric reactivity is also limited.

At present, there are many methods for evaluating volume responsiveness. Common and popular methods include general evaluation, passive leg raising test, echocardiography (color Doppler technique), continuous cardiac output monitoring with pulse indication, etc. 38. There are invasive methods and non-invasive methods. However, there is no more ideal method, and it is still difficult to implement effective fluid resuscitation in patients with septic shock. Traditional assessment indicators, such as CVP, are widely used to evaluate effective circulating blood volume and guide fluid therapy because of their easy availability 39-40. During breathing, the intrathoracic pressure will rise and fall periodically, resulting in a decrease and increase in the blood return to the heart, and the diameter of the inferior vena cava changes accordingly 41-42.

In the presence of reduced effective circulating blood volume, the diameter of the inferior vena cava decreases, while the diameter of the VIVC increases. Therefore, VIVC can be used to predict volume response and guide fluid resuscitation therapy 43. The non-invasiveness, simplicity, and reproducibility of ultrasound measurements of the inferior vena cava's respiratory variability index 44. Some scholars believe that VIVC can also be used as a potential indicator for evaluating volume resuscitation in patients with septic shock, and the VIVC measurement method is simple and non-invasive, which is convenient for clinical application 45. The results of this study indicated that there exhibited no difference in 6h CVP after fluid resuscitation. The CVP of the study group was higher at 12h and 24h after fluid resuscitation, and the VIVC level of the study group at 6h, 12h and 24h after fluid resuscitation was higher. Excessive fluid resuscitation in patients in septic shock is more likely to increase CVP and VIVC, leading to a range of pathophysiological changes such as reduced cardiac output, impaired venous return, reduced blood pressure and increased pulmonary artery pressure, and even to organ dysfunction 46-47.

This study further analysed the predictive value of CVP combined with VIVC on fluid resuscitation volume responsiveness in patients with septic shock. The results indicated that the Pearson correlation analysis indicated that the levels of CVP and VIVC were noticeably correlated with 24-hour fluid resuscitation in patients with septic shock. In clinical practice, it is necessary to pay attention to grasp its internal relationship, and further study its internal relationship. Multivariate logistic regression analysis indicated that CVP and VIVC were risk factors for fluid resuscitation in patients with septic shock. The AUC of ROC of CVP for predicting fluid resuscitation in patients with septic shock was 0.694 and the cut-off value was 0.932, the sensitivity was 46.9%, and the specificity was 87.5%. The AUC of VIVC for predicting fluid resuscitation in patients in septic shock was 0.776 with a cut-off value of 0.688, a sensitivity of 50.0% and a specificity of 90.0%. The AUC for CVP+VIVC to predict fluid resuscitation in patients with septic shock was 0.948, with a critical value of 1.420, sensitivity of 90.6% and specificity of 87.5%. It can further suggest that CVP combined with VIVC has high clinical value in predicting the prognosis of septic shock patients and can be widely used in clinical practice. Real-time monitoring of CVP and VIVC levels in patients with septic shock and timely intervention and correction can effectively enhance the prognosis of patients with septic shock 48-49.

To the best of our knowledge, composite biomarkers can over-fit the current data, so our current study also has the limitation of lacking external validation. The potential future study could be by combining the resulting indices in an ensemble machine learning model, a strategy that incorporates advanced techniques in artificial intelligence that may significantly improve diagnostic performance 50.

To sum up, CVP+VIVC may play a good role in the evaluation of volume response in patients with septic shock, and it is better than single CVP and VIVC. CVP+VIVC can be used to predict the volume response of fluid resuscitation in patients with septic shock and is of great significance in guiding clinical fluid resuscitation therapy.
